# Prevalence of clinical findings at examinations of young Swedish warmblood riding horses

**DOI:** 10.1186/1751-0147-55-34

**Published:** 2013-04-18

**Authors:** Lina Jönsson, Lars Roepstorff, Agneta Egenvall, Anna Näsholm, Göran Dalin, Jan Philipsson

**Affiliations:** 1 Department of Animal Breeding and Genetics, Uppsala PO Box 7023 Sweden; 2 Department of Equine Studies, Uppsala PO Box 7043 Sweden; 3 3Department of Clinical Sciences, Swedish University of Agricultural Sciences, PO Box 7054 750 07, Uppsala, Sweden

**Keywords:** Medical health, Orthopaedic health, Flexion test, Hoof, Clinical signs

## Abstract

**Background:**

Soundness of an individual horse is important for animal welfare and owner economy. However, knowledge of health status in normal horse populations is limited due to lack of systematic health recordings. The aim of the investigation was to study the prevalence of veterinary clinical findings in 4-5-year-old Swedish warmblood riding horses, and their influence on overall health scores, where associations to future longevity has been indicated.

**Results:**

The prevalence of clinical findings in 8,281 horses examined during 1983–2005 was studied according to a standardised protocol and related to overall health scores in linear statistical models. Effects of sex, age, examination event and changes over time were included. In total, 49% of the horses had clinical findings of medical health (MED), 42% in hooves (HOOF) and 74% of palpatory orthopaedic health (PALP). However, only 6%, 3% and 24% had moderate or severe findings, of MED, HOOF and PALP, respectively. Flexion test reactions were reported in 21% of the horses (5% moderate/severe), heavily influencing the overall score (H2). One fifth of these horses also had findings of unprovoked lameness while 83% had PALP findings (44% with moderate/severe findings). Acute clinical signs, i.e. heat or soreness, had a large influence on the H2 score but were rare, whereas more common clinical findings had smaller effects on overall health. Large variations in recorded health results were observed among events. A decrease in findings has occurred since 1983, in particular for PALP findings.

**Conclusions:**

Results of occurrence and relevance of evaluated clinical findings could be used for advice on preventive actions to keep horses sound, and possibly for benchmarking, and genetic evaluation of health traits. The distinct effect of event on recorded clinical findings emphasises that further harmonisation of veterinary examinations are desirable.

## Introduction

Musculoskeletal disorders are the predominant cause of culling horses. In Sweden between 55 and 70% of cullings [1,2] and in the German Warmblood 61% of insurance claims concerned movement related diseases [3]. Further, 60% of former Hanoverian auction horses had at least one period of lameness causing a relevant interruption in training, and 35% had multiple periods of lameness in the years following the auction [4]. At the same time, soundness is the most important trait when marketing a horse [5]. Only if sound, talents for performance are considered. Thus, health status is highly important as it affects animal welfare and the utility and saleability of the horse. However, reports regarding prevalence of clinical findings in normal horse populations are scarce. Thus, it is of great interest to map the general health status of the sport horse.

The Swedish Riding Horse Quality Test (RHQT) was introduced in 1973 and includes evaluations of health, conformation, gaits and jumping. The RHQT includes broken-in 4-year-old warmblood riding horses of both genders and 5-year-old mares that had a foal the previous year. The test is designed to fit all riding horses regardless of talent and no prior qualifications are required. The young age of participating horses suggests a limited environmental effect of training on health results. Test locations are distributed throughout the country. The RHQT performance scores have been found to be good early predictors of future performance and to function as basis for breeding value estimation [6]. In 1973–1986, low overall health scores at the RHQT were significantly related to early future culling (n = 1,815) [7]. In 1994 the health examination was considered an important motive for participation at the RHQT by 66% of participants. Further, the health examination obtained a score of 4.3 out of 5 regarding fulfillment of its objective [8]. However, specific health information, besides the overall scores, has previously not been evaluated.

The aim of the investigation was to study the prevalence of clinical findings and their influence on the overall assessment of health made by examining veterinarians in an extensively recorded population of young sport horses.

## Material and methods

### Material

**Figure 1:**
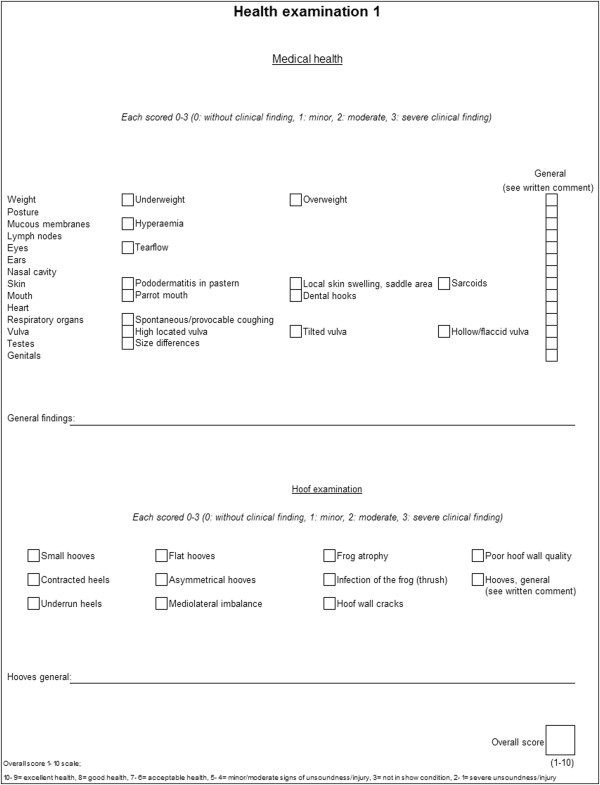
**Protocol used for health examination 1.** Including separate clinical findings examined for and scale of scoring.

**Figure 2:**
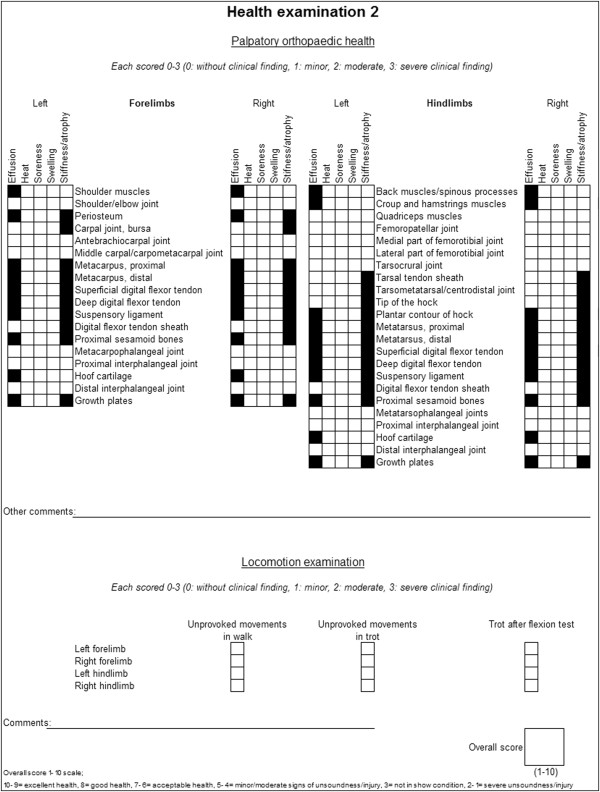
**Protocol used for health examination 2.** Including separate clinical findings examined for and scale of scoring.

Health information was obtained from RHQT events for horses eligible for the Swedish Warmblood (SWB) studbook during 1983–1984 and 1988–2005. Data after that period was not included as the examination regime was slightly changed. The data from 1985–1987 had not been saved. For protocols of health examinations see Figures 1 and 2, respectively. All horses at an event were examined in the same environment by one veterinarian for medical health (MED) and hoof shape & quality (HOOF), constituting health examination 1. Horses were examined by another veterinarian for palpatory orthopaedic health (PALP) and locomotion including flexion tests (LOCO), constituting health examination 2. The examinations targeted clinical findings in specified anatomical locations of the horse, which together represent overall health status. Specific clinical findings were scored 0–3 (0: no clinical finding, 1: minor, 2: moderate or 3 severe clinical finding). Further, overall health scores (H1 and H2) between 1 (very poor) and 10 (excellent) were given by each examiner. Examiners were experienced horse practitioners with compulsory training in judging regimes of the RHQT. Examinations were performed using palpation, auscultation with stethoscopes and flexion tests. All horses were documented for health status on identical protocols. Opportunities were given to include free text comments but were mainly used for clarification.

### Method and data structure

Available paper protocols of 9,053 horses were scanned to a digital picture, then digitally read to classify contents using the neural network toolbox in Matlab®^a^, followed by manual validation. Identity was confirmed in the SWB database. In total, 8,281 horses had complete health examinations and a confirmed identity. In the available data most horses were 4 years old (n = 7,788), and 493 were 5 years old. Data represented 3,879 mares and 4,402 males (predominantly geldings). Each year 4–14 events were included, generating 193 events, each with 4 to 129 evaluated horses. At these events 57 and 54 different veterinarians were employed for health examination 1 and 2 respectively. Approximately, a random half of conducted events were included in the study. During the years 1979–2001 a total of 98 000 SWBs were born. If assuming 7% loss of horses each year due to death, export etc. [1], 23% of available horses participated in the RHQT during 1983–2005. The 8,281 studied horses represented 53% of tested horses in that time period.

### Statistical analysis

Descriptive statistics and statistical analyses were performed using the statistical package SAS®^b^. The overall health status of MED, HOOF, PALP and LOCO, respectively, was studied as the sum of clinical findings, including severity (0–3), at each type of examination. Thus, the sum of clinical findings increased both with number and severity of findings. Summed values included both bilateral clinical findings. Corresponding summation of PALP findings was performed in 4 groups of systemic location, i.e. joints, muscles, tendons & suspensory ligaments, and skeleton & hoof cartilage and into 5 groups of clinical sign i.e. effusion, heat, soreness, swelling, stiffness/atrophy, irrespective of whether findings were bilateral or not. Clinical signs were also grouped into primarily acute (heat, soreness) or primarily chronic (effusion, swelling and stiffness/atrophy) findings.

High overall H1 and H2 scores were used frequently resulting in skewed distributions (Additional file 1). However, data were kept untransformed in subsequent analyses because extensive transformation trials showed similar fixed effect results and distribution of residual values.

Fixed effects of age, sex and event on health status results were analysed using General Linear Models (GLM). Effects of separate clinical findings on H1 or H2 were estimated as class effects of ‘minor’ respectively ‘moderate or severe’ finding compared to no finding using GLM. The employed model included the fixed effects of age, sex, event, and clinical finding. The separate clinical findings were included one at a time and therefore 39 analyses were performed for health examination 1 and 123 analyses for health examination 2. Non-used observations were excluded and right and left observations were pooled irrespectively of whether findings were bilateral or not. Differences in H2 scores between horses with different categories of clinical findings were evaluated with significance tests of differences between least squares means using GLM.

## Results

**Figure 3:**
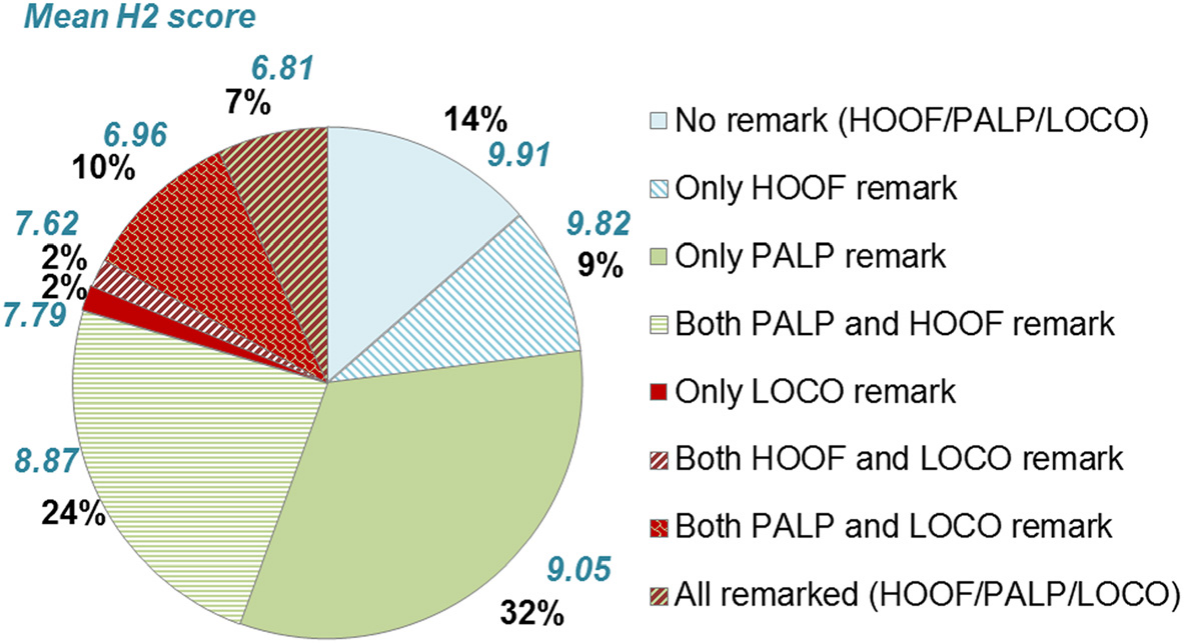
**Proportion of horses with clinical findings in one or several types of examination.** Hoof examination (HOOF) in stripe pattern (total 42%), palpatory orthopaedic examination (PALP) in green shades (total 74%), and locomotion examination (LOCO) in red shades (total 21%), ordered after mean orthopaedic health score (H2) read clockwise, n = 8,281.

In total, 49% of studied horses had clinical findings of MED, 42% of HOOF and 74% of PALP. However, only 6%, 3% and 24% had moderate or severe findings of MED, HOOF and PALP, respectively. Flexion test reactions were reported in 21% of horses (5% moderate/severe). The proportions of horses with clinical findings in HOOF, PALP and/or LOCO examinations are shown in Figure 3, accompanied by mean H2 scores. The H2 score gradually decreased as the number of examination types with reported clinical findings increased, where LOCO had the largest separate influence. Differences in H2 scores were significant (p < 0.05) between all groups, except for the difference between ‘no findings’ vs. ‘only HOOF findings’ and between ‘only LOCO findings’ vs. ‘HOOF and LOCO findings’. Indicating that, for example if findings occur both during PALP and LOCO examinations, this results in a significantly lower H2 score compared to horses with only findings within the LOCO examination. Generally, H2 scores under 8 were found in horses with LOCO findings. Clinical findings of MED were present in 40-60% of horses in each group in Figure 3 (results not shown). Additional file 1 shows that most horses (84%) were characterised as healthy regarding the H2 score (score 8–10) and the proportion was even higher for H1 scores (97%). Mean H1 and H2 score was 9.42 and 8.78, with medians of 10 and 9 respectively.

Clinical findings occurring in ≥ 2% of examined horses within health examination 1 and 2 are presented in Table 1. Within health examination 1 clinical skin and mouth findings were the most frequent MED findings. The most common clinical HOOF findings concerned hoof wall quality. Findings of hoof size and shape were also quite common with emphasis on asymmetrical, small and flat hooves. Among horses with clinical MED findings the mean sum of findings, including severity, was 1.7 with a maximum of 9. The corresponding results for HOOF were 1.9 and 7. For health examination 2 most common clinical PALP findings generally concerned effusions or swellings in joints, accompanied by flexion test reactions.

**Table 1:** Most frequent clinical findings (≥2%) during health examination 1 and 2, and their estimated effect on respective overall score (H1/H2)

	**Clinical finding (%)**				**Effect of finding on overall score**				
					**Minor**		**Moderate or severe**		
	**Minor**	**Moderate**	**Severe**	**Total**	**Estimate**^**1**^	***p=***	**Estimate**^**1**^	***p=***	**R**^**2**^**(%)**^**2**^
**Health examination 1**									
*Medical health*									
Skin, general	13.4	0.4	0.1	**14.0**	−0.24	***	−1.07	***	1.60
Mouth general (e.g. wolf teeth) ^3^	11.2	0.2	0.0	**11.4**	0.02	ns.	−0.15	ns.	0.01
Local skin swelling in saddle area	7.9	1.5	0.1	**9.5**	−0.46	***	−0.89	***	3.56
Pododermatitis in pastern (mud fever)	6.1	1.2	0.2	**7.5**	−0.40	***	−1.15	***	3.61
Parrot mouth ^3^	5.7	0.6	0.1	**6.3**	−0.11	**	−0.51	***	0.30
Lymph nodes, general	3.6	0.7	0.0	***4.3***	−0.66	***	−1.03	***	1.86
Skin sarcoids	3.2	0.4	0.0	***3.5***	−0.47	***	−0.90	***	1.36
Mucous membrane hyperaemia	2.6	0.1	0.0	***2.7***	−0.11	*	−0.28	ns.	0.04
Dental hooks ^3^	2.2	0.2	0.0	***2.4***	−0.16	**	−0.56	**	0.14
Tilted vulva ^4^	2.3	0.1	0.0	***2.4***	0.06	ns.	−0.39	ns.	0.02
Hollow/flaccid vulva ^4^	2.0	0.2	0.0	***2.2***	−0.14	ns.	−0.32	ns.	0.07
Overweight	1.9	0.1	0.0	***2.0***	−0.02	ns.	−0.13	ns.	0.00
*Hoof examination*									
Hoof wall cracks	11.1	0.6	0.0	**11.7**	−0.56	***	−0.90	***	4.33
Hooves, general	10.5	0.2	0.0	**10.7**	−0.23	***	−0.74	***	0.76
Asymmetrical hooves	6.8	0.2	0.0	**7.1**	−0.24	***	−1.04	***	0.74
Small hooves	6.3	0.3	0.0	**6.6**	−0.07	*	−0.04	ns.	0.03
Flat hooves	6.2	0.4	0.0	**6.6**	−0.47	***	−0.88	***	1.90
Underrun heels	5.2	0.2	0.0	***5.4***	−0.35	***	−1.26	***	1.32
Infection of the frog (thrush)	2.9	0.4	0.0	***3.4***	−0.39	***	−1.28	***	0.90
Contracted heels	3.3	0.1	0.0	***3.4***	−0.68	***	−1.00	***	2.08
Mediolateral imbalance	2.9	0.1	0.0	***3.0***	−0.38	***	−1.30	***	0.75
Poor hoof wall quality	2.6	0.2	0.0	***2.8***	−0.79	***	−1.60	***	2.68
**Health examination 2**									
*Palpatory orthopaedic health*									
***Effusion***									
Hindlimb digital flexor tendon sheath	23.5	2.8	0.3	**26.7**	−0.17	***	−0.88	***	1.20
Tarsocrural joint	13.6	3.7	0.6	**17.9**	−0.40	***	−0.95	***	2.39
Metatarsophalangeal joint	12.1	3.3	0.5	**15.9**	−0.47	***	−1.09	***	2.66
Middle carpal/carpometacarpal joint	8.8	3.9	0.5	**13.2**	−0.48	***	−1.10	***	2.81
Metacarpophalangeal joint	7.3	2.6	0.2	**10.2**	−0.60	***	−1.23	***	2.76
Femoropatellar joint	4.2	3.5	0.6	**8.3**	−0.30	***	−1.17	***	2.19
Forelimb digital flexor tendon sheath	3.2	0.5	0.0	**3.7**	−0.40	***	−1.03	***	0.49
Forelimb distal interphalangeal joint	3.0	0.5	0.0	**3.5**	−0.42	***	−1.10	***	0.56
Medial part of femorotibial joint	1.7	0.5	0.1	**2.3**	−0.55	***	−1.07	***	0.56
***Swelling***									
Metacarpus, proximal	12.6	3.0	0.4	**16.0**	−0.12	**	−0.46	***	0.41
Metatarsus, proximal	5.1	1.0	0.3	**6.4**	−0.03	ns.	−0.38	**	0.10
Metacarpus, distal	3.0	0.6	0.1	**3.7**	−0.16	*	−0.24	ns.	0.06
Metatarsus, distal	2.5	0.6	0.1	**3.2**	−0.15	ns.	−0.77	***	0.25
Metacarpophalangeal joint	1.6	1.1	0.2	**2.9**	−0.27	***	−1.06	***	0.75
Metatarsophalangeal joint	1.8	0.7	0.3	**2.8**	−0.24	*	−0.83	***	0.40
Tarsometatarsal/centrodistal joint	1.8	0.7	0.2	**2.6**	−0.49	***	−1.57	***	1.18
Tip of the hock	1.9	0.5	0.1	**2.5**	−0.41	*	−1.01	***	0.17
***Atrophy/Stiffness***									
Croup/hamstrings muscles	3.3	0.8	0.1	**4.2**	−0.92	***	−1.69	***	2.43
Quadriceps muscles	1.6	0.4	0.1	**2.1**	−0.86	***	−1.62	***	1.21
***Soreness***									
Back muscle/spinous processes	2.9	0.6	0.0	**3.5**	−0.76	***	−1.68	***	1.63
*Locomotion examination*									
Hindlimb post flexion test movements in trot^5^	10.2	2.4	0.5	**13.1**	−1.73	***	−3.05	***	11.50
Forelimb post flexion test movements in trot^5^	9.0	1.7	0.2	**10.8**	−1.78	***	−3.10	***	8.97
Hindlimb unprovoked movements in trot	2.4	0.2	0.0	**2.7**	−2.06	***	−3.61	***	6.34

^1^Estimate of class effect on overall score compared to no finding, from single trait analyses.

^2^R^2^ values from single trait analysis when the summed R^2^ from fixed effects has been removed (35% for H1 and 27% for H2).

^3^Missing information for 7 horses, n = 8,274.

^4^Out of 3879 examined mares.

^5^Missing information for 26 horses, n = 8,255.

ns = non sign* = p < 0.05** = p < 0.01*** = p < 0.001.

Clinical finding (%) indicates prevalence of findings. N = 8,281 examined horses if nothing else indicated.

### Locomotion examination (LOCO)

**Figure 4:**
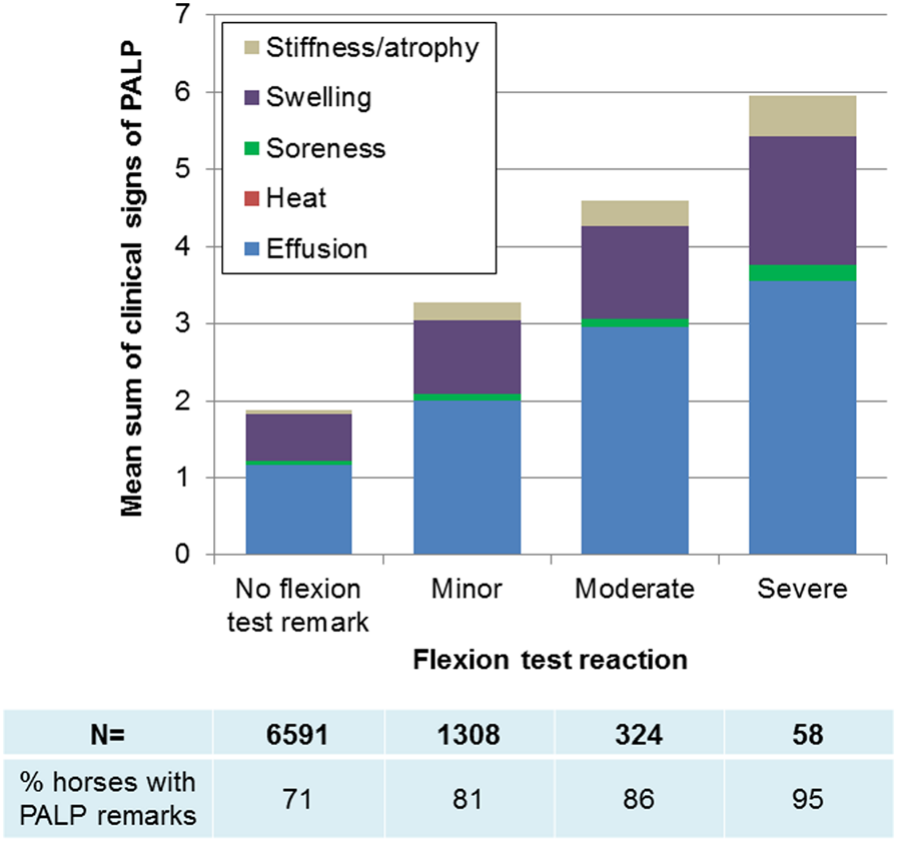
Mean sum of palpatory orthopaedic health (PALP) findings, including severity, in horses with different degrees of flexion test reactions.

Few horses showed signs of lameness in unprovoked walk (1%) and trot (4%), including both fore- and hindlimbs. However, flexion test reactions were found in 11% and 13% of horses in fore- and hindlimbs, respectively. Among horses with flexion test reactions, 20% showed unprovoked lameness. The highest sum of LOCO findings, including severity, was 21 with a mean of 2.0 among horses with LOCO findings. As seen in Figure 4 the number and severity of PALP findings increased with increasing degree of flexion test reactions, for all types of clinical signs.

### Palpatory orthopaedic examination (PALP)

**Figure 5:**
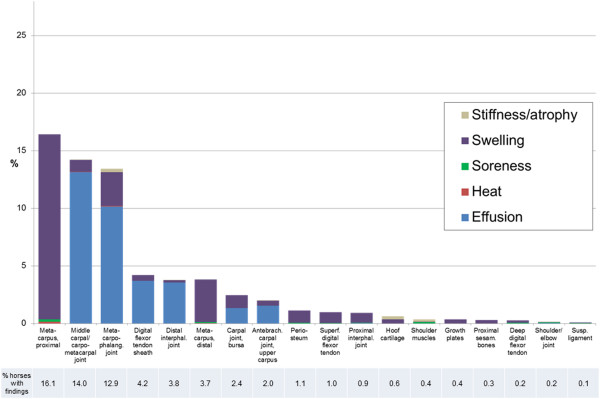
**Prevalence of clinical signs of palpatory orthopaedic health (PALP) in examined forelimb/shoulder locations.** Total prevalence of horses with clinical findings at respective location presented below.

**Figure 6:**
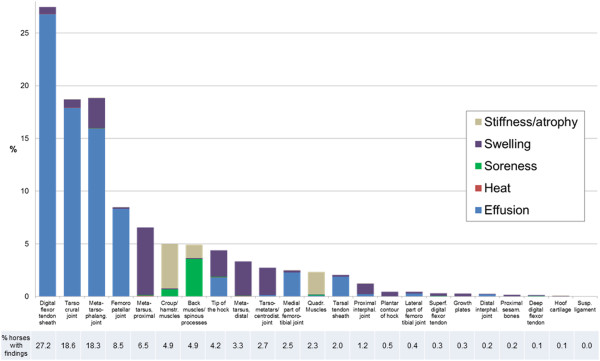
**Prevalence of clinical signs of palpatory orthopaedic health (PALP) in examined hindlimb/croup locations.** Total prevalence of horses with clinical findings at respective location presented below.

The prevalence of each clinical sign of PALP in fore- and hindlimbs are presented in Figures 5 and 6, together with the total percentage of examined horses with findings at each location. Generally, more findings were reported in hindlimbs. Additional analyses (results not shown) showed that bilateral findings (all clinical signs included) were generally found in 30% of horses with findings at each examined location. Locations with >50% bilateral findings in hindlimbs/croup were; back muscles/spinous processes (81%), digital flexor tendon sheaths (78%), growth plates (65%), metatarsophalangeal joints (62%) and hoof cartilage (60%). Corresponding forelimb/shoulder locations were; growth plates (83%), distal interphalangeal joint (65%), metacarpophalangeal joints (61%), digital flexor tendon sheaths (58%) and shoulder muscles (57%). In examined horses most clinical findings were evenly distributed between left and right side at each location (results not shown). Among horses with PALP findings the mean sum of findings including severity was 4.4 with a maximum of 36. Further, 98% and 7% of horses with PALP findings had chronic and acute findings respectively; 5% showed signs of both categories.

### Effects of fixed factors

A significant effect of event (p < 0.0001) and gender (p < 0.05) was present in all types of examination where mares had slightly lower overall scores and more clinical findings, except for HOOF, where no significant gender differences were found. The age effect was non-significant for types of health examination but significant (p < 0.05) for clinical signs of soreness where 5-year-old mares had more findings, in particular for soreness in back muscles/spinous processes. According to the coefficient of determination (R^2^), fixed effects explained 35% of H1 and 27% of H2 variation, primarily due to variation among events.

### Effects of specific clinical findings on overall scores

Clinical findings with the largest effect on overall scores (≤ −1.5 points) are found in Table 2, ordered by moderate/severe finding effects on overall health scores (H1/H2) within each group of examination. In particular regarding effects on H2 scores several rare but acute PALP findings were found to have the largest influence. Additional analyses revealed that the H2 score decreased significantly between horses with ’no finding’, ‘chronic finding’, ‘acute finding’ and ‘both acute & chronic finding’ respectively, in decreasing order (p < 0.05 between all groups). Further, LOCO findings were found to have a large influence on the H2 score. Among horses with any type of flexion test reaction 36% obtained an H2 score of ≤6 with a mean of 7.0, compared to a mean of 9.2 among horses without flexion test reactions, p < 0.0001. For horses with severe flexion test reactions 91% had a score of ≤6 (mean: 4.8).

**Table 2:** Clinical findings with largest effect (≤ −1.5 points) on respective overall score (H1/H2) at health examinations 1 and 2

	**Effect of clinical finding on overall score**						
	**Minor**			**Moderate or severe**			
	**Estimate**^**1**^	***p=***	**Prevalence (%)**	**Estimate**^**1**^	***p=***	**Prevalence (%)**	**R**^**2**^**(%)**^**2**^
**Health examination 1** *Medical health*							
Spontaneous or provocable cough	−1.16	***	1.20	−2.04	***	0.20	2.80
Heart, general	−1.05	***	0.90	−1.93	***	0.02	1.50
Nasal cavity, general	−0.24	**	1.30	−1.76	***	0.05	0.27
*Hoof examination*							
Poor hoof wall quality	−0.79	***	2.60	−1.60	***	0.20	2.68
**Health examination 2** *Palpatory orthopaedic health*							
***Soreness***							
Tarsocrural joint	.	.	0.00	−6.72	***	0.01	0.30
Hindlimb superficial digital flexor tendon	−0.01	ns.	0.02	−6.49	***	0.01	0.30
Forelimb superficial digital flexor tendon	−1.27	ns.	0.04	−6.49	***	0.01	0.30
Hindlimb deep digital flexor tendon	−1.17	ns.	0.01	−6.49	***	0.01	0.30
Forelimb deep digital flexor tendon	−2.38	**	0.02	−4.08	***	0.02	0.30
Quadriceps muscles	−0.07	ns.	0.07	−2.08	ns.	0.01	0.03
Shoulder/elbow joint	−1.15	ns.	0.05	−1.83	ns.	0.01	0.05
Back muscles/spinous processes	−0.76	***	2.93	−1.68	***	0.60	1.63
***Heat***							
Tarsocrural joint	2.04	ns.	0.01	−5.06	***	0.01	0.20
Metacarpus, proximal	−0.19	ns.	0.13	−3.07	*	0.01	0.10
Forelimb deep digital flexor tendon	.	.	0.00	−1.71	ns.	0.01	0.02
Metacarpophalangeal joint	−0.66	ns.	0.02	−1.50	ns.	0.02	0.03
***Effusion***							
Shoulder/elbow joint	1.21	ns.	0.02	−4.61	***	0.01	0.20
Tarsometatarsal/centrodistal joint	−0.70	ns.	0.11	−1.55	*	0.04	0.07
***Stiffness/Atrophy***							
Hindlimb pastern joint	0.71	ns.	0.01	−4.03	***	0.01	0.10
Femoropatellar joint	−1.49	ns.	0.01	−2.43	*	0.01	0.05
Medial part of femorotibial joint	.	.	0.00	−2.43	*	0.01	0.04
Middle carpal/carpometacarpal joint	−1.21	ns.	0.01	−2.34	*	0.01	0.04
Shoulder/elbow joint	0.83	ns.	0.02	−2.11	ns.	0.01	0.04
Metacarpophalangeal joint	−0.21	ns.	0.24	−1.90	***	0.06	0.12
Forelimb hoof cartilage	−0.27	ns.	0.25	−1.77	ns.	0.01	0.03
Croup/hamstrings muscles	−0.92	***	3.33	−1.69	***	0.86	2.43
Quadriceps muscles	−0.86	***	1.63	−1.62	***	0.51	1.21
***Swelling***							
Back muscles/spinous processes	−0.90	ns.	0.06	−3.40	***	0.04	0.20
Forelimb hoof cartilage	−0.44	ns.	0.29	−2.50	***	0.06	0.23
Tarsometatarsal/centrodistal joint	−0.49	***	1.76	−1.57	***	0.81	1.18
*Locomotion examination*							
Forelimb unprovoked movements in trot	−1.89	***	1.47	−4.27	***	0.09	3.30
Hindlimb unprovoked movements in trot	−2.06	***	2.40	−3.61	***	0.27	6.34
Forelimb post flexion test movements in trot^3^	−1.78	***	8.96	−3.10	***	1.90	8.97
Hindlimb post flexion test movements in trot^3^	−1.73	***	10.20	−3.05	***	2.94	11.50
Forelimb unprovoked movements in walk	−0.61	**	0.43	−3.02	***	0.03	0.20
Hindlimb unprovoked movements in walk	−1.50	***	0.60	−1.97	***	0.06	0.77

^1^Estimate of class effect on overall score compared to no finding, from single trait analyses.

^2^R^2^ values from single trait analysis when the summed R^2^ from fixed effects has been removed (35% for H1 and 27% for H2).

^3^Missing information for 26 horses, n = 8,255.

ns = non sign* = p < 0.05** = p < 0.01*** = p < 0.001.

Ordered after decreasing magnitude of estimates for moderate/severe findings within examination groups. N = 8,281 if nothing else indicated.

### Synergy effects of PALP and LOCO findings

Within horses showing flexion test reactions 83% had PALP findings (44% moderate/severe findings). The prevalence of LOCO findings increased among horses with acute PALP findings (30%) compared to horses with chronic findings (23%) and horses without PALP findings (13%). The most common PALP finding among horses with flexion test reactions were effusions in the digital flexor tendon sheath in hindlimbs (25%), metacarpo- and metatarsophalangeal joints (18% and 24%, respectively), tarsocrural joint (23%), middle/lower part of carpus, i.e. middle carpal joint and carpometacarpal joint (21%) and femoropatellar joint (17%), swelling in proximal part of metacarpus (16%), atrophied croup/hamstrings muscles (11%), atrophied quadriceps muscles (7%), and swelling in tarsometatarsal/centrodistal joint (7%). All mentioned PALP prevalences were significantly elevated compared to horses without LOCO findings, except for effusion in the hindlimb digital flexor tendon sheaths and swelling in proximal part of metacarpus. Soreness in back muscles/spinous processes was elevated to 5% in horses with LOCO findings.

### Common clinical findings among different overall scores

**Figure 7:**
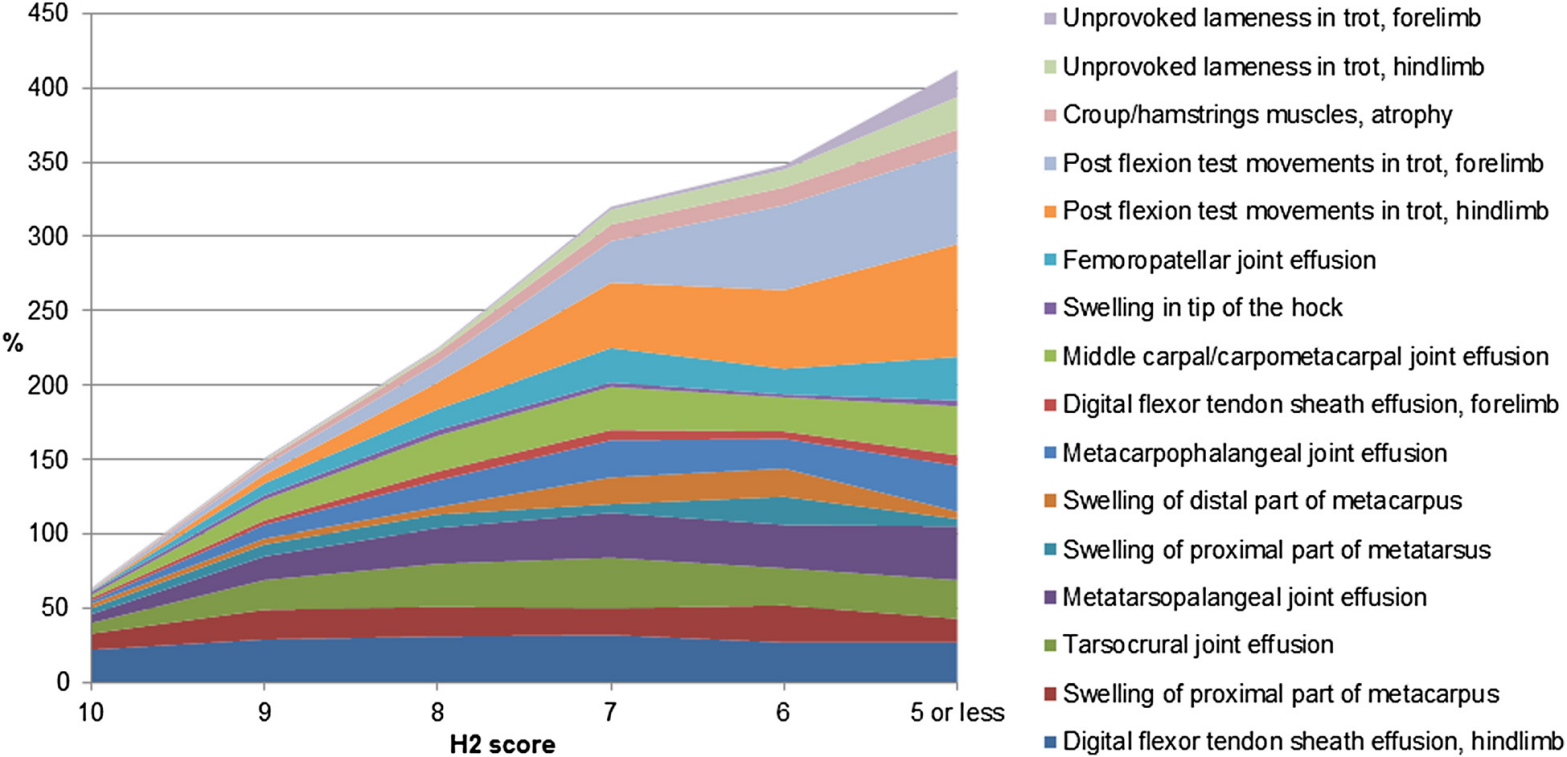
**Variation in extent of clinical findings between horses with different orthopaedic health scores (H2).** Including the 10 most common findings associated to each H2 score (16 findings in total) of palpatory orthopaedic health (PALP) and locomotion (LOCO).

**Figure 8:**
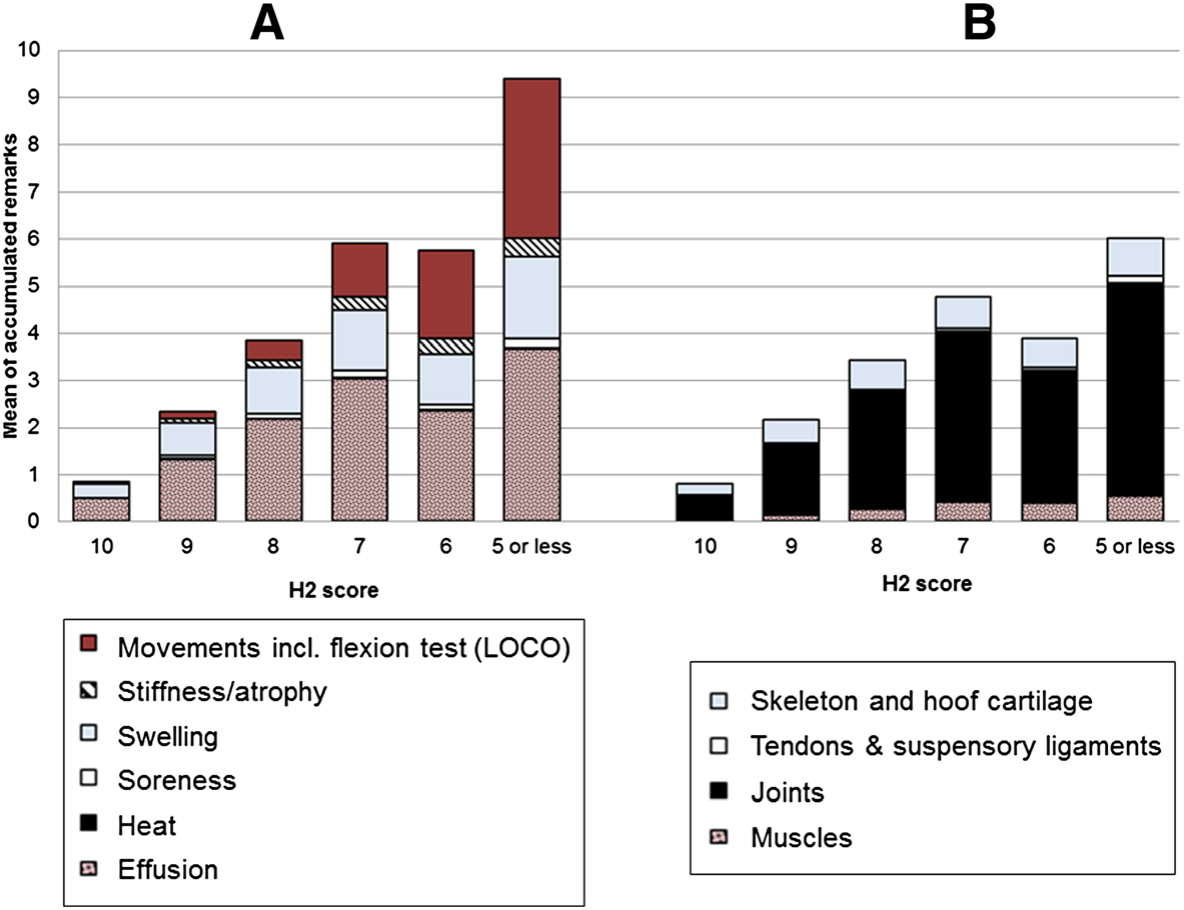
**Mean sum of clinical findings, including severity, of clinical signs (A) and systemic locations (B) of palpatory orthopaedic health (PALP).** Results compare horses with each orthopaedic health score (H2), diagram **A** is complemented with locomotion examination (LOCO) results.

Figure 7 illustrates percentages of horses with clinical findings within each H2 score, among the top 10 most common findings associated to one or several H2 scores (16 findings in total), from PALP and LOCO examinations. A steady overall increase in percent horses with clinical findings was seen as the H2 score decreased, which was also true for a majority of presented individual clinical findings. An exception was effusion in the hindlimb digital flexor tendon sheaths that were similar for all H2 scores. A slight trend deviation was seen for horses with H2 score ≤5 and 6 where some PALP findings decreased simultaneously as LOCO findings increased, indicating LOCO status to have a distinct effect on H2 regardless of PALP status. The most frequent clinical finding of horses with H2 scores ≤5 that increased compared to other H2 scores were: flexion test reactions (fore- and hindlimbs), effusions in metacarpo- and metatarsophalangeal joints, middle/lower part of carpus, femoropatellar- and tarsocrural joint and unprovoked lameness in trot. The overall results of over 100% mean that several clinical findings generally were present in each horse. The same trend of increase in findings with decreasing H2 score was seen when including all PALP and LOCO findings and their rate of severity (Figure 8). This figure also shows that effusions were the most common clinical sign regardless of given H2 score, followed by LOCO findings that both increased with decreasing H2 score. Further, most PALP findings were found at joint related locations. Generally, less than one minor finding was present in horses with H2 score 10 and horses with score 9 had one moderate (or two minor) finding(s). The same relationship was found between H1 scores and health examination 1 findings (results not shown). Clinical findings of HOOF and MED increased equally with decreasing H1 score, where horses with H1 score ≤5 generally had more signs of inflammation.

### Trend in clinical findings and overall health scores

A decreasing trend in sum of PALP findings (including severity) was seen during the test period (Additional file 2). Similar trends, but not as distinct, appear for MED, HOOF and LOCO. In the beginning of the 1990’s H1 and H2 scores generally increased, which to some extent was confirmed by a decrease in sum of clinical findings during the same time, in particular for PALP.

## Discussion

Studies on equine population health are scarce as systematic health recording is not common practice in the horse industry. The regime of milk cow health recording in Sweden and some other countries has proved extremely useful for improving general population health and longevity. In contrast, the lack of systematic health registration in horses has limited the knowledge of health status in horse populations, and genetic evaluation of predisposition for unsoundness is absent. The usefulness of systematic health recording of equine hospital data was also demonstrated regarding studies of osteochondrosis (OC) [9]. Health recordings from the RHQT are globally unique as a large number of health traits have been systematically recorded during several years. Presented results expectedly represent the average young riding horse population in Sweden fairly well, with the exception that the worst cases will likely not participate if owners are aware of the unsoundness, e.g. lame horses. Failure to participate because of physical problems at such a young age is likely to be due to acute injuries or contagious diseases, which are expected to occur randomly.

Equine health status has previously mostly been studied on smaller data sets, or on data diverging from the typical riding horse population by e.g. old age, health status or selection for talent prior to auctions [4,10]. Present results generally show lower prevalences compared to studies of older horses, which is expected as clinical findings commonly increase with age [10,11]. Further, differences may be due to variation among examiners or populations. The number and severity of clinical findings has generally decreased over the years, both within examiner (results not shown) and overall. This might represent a combination of better general health in the population and improved health care. Also, increased owner awareness over time, might have influenced towards a lower participation rate in horses with substantial health lesions. However, a recent British study found that generally horse owner awareness of health abnormalities in their horses is low compared to veterinary examination results [12].

The large extent of reported clinical findings (Figure 3) in relation to mean H2 scores indicates that examiners aimed to include every finding in the protocol, also minor ones. Generally, overall scores were high suggesting findings to have a small assessed effect on overall health, at this young age. An important distinction was made between recording findings as objectively as possible on one hand, and on the other hand assessing overall health status into an overall score. Both have a unique informative value. According to Tables 1 and 2, findings with the largest separate effects on overall health status i.e. findings of soreness or heat, were often serious in character but rare in the population, whereas more frequently occurring findings i.e. effusions and swelling, had a smaller individual effect. During health examination 2 only 6 separate clinical findings both had a prevalence ≥ 2% and an effect of moderate/severe findings of −1.5 points or stronger: flexion test reactions in both fore- and hindlimbs, sore back muscles/spinous processes, atrophied croup/hamstrings- and quadriceps muscles, and swelling in tarsometatarsal/centrodistal joint. For health examination 1 only findings of poor hoof wall quality met the same criteria. These findings might represent what is most important to consider in the future, if only focusing on separate clinical findings.

The effect of event was highly significant on health results, which is also true for RQHT performance results [6], and for examiner effect in other health studies [11,13,14]. Factors, apart from examiner, included in the event effect were e.g. examination site and time dependent changes. Events were held within a few weeks in early autumn each year, thus season was quite equal between events, apart from geographical differences. A substantial part of the event effect was due to examiner, which had a significant effect on its own in separate analyses (results not shown), however, not explaining all event variation. RHQT veterinarians attend compulsory training to standardise their examinations, but present results suggest that further efforts are needed. In the meantime means to analytically correct examination results for examiner are necessary. In present analyses all systematic differences between events, including effects of examiner, have been adjusted for.

A desirable improvement in the studied recording scheme would be to exclude HOOF from the H1 score, and rather keep it separate. Originally, HOOF was included within the H1 examination in order for both examinations to take equally long time. Horses with clinical hoof findings obtained slightly lower H2 scores, even if HOOF was not included in that examination (significant differences in 2 out of 4 groups, Figure 3). Radiographic equipment or endoscope was not used as the horses, usually about 40, have to complete the test in one day. Also, horses are judged for performance shortly after the veterinary examination and cannot be sedated. The effect of rider was not accounted for in this study. A survey among RHQT participants has shown that rider experience of broken-in horses significantly influenced the H2 score [8]. However, a majority of riders only have one horse, thus the effects of rider and horse, respectively, are usually confounded.

Analysis of a limited RHQT data set showed that horses with H2 score ≤5 generally had twice as high risk of early culling compared to those with score ≥9 [7]. The present study could further reveal that the most common separate findings that increased in horses with H2 ≤5 were LOCO results and effusions in metacarpo-/metatarsophalangeal joints and in middle/lower part of carpus, tarsocrural- and femoropatellar joints. This information may be used in veterinary practice when assessing relevance of clinical findings or for future preventive actions. Commonly affected locations coincide well with statistics of insurance claims for morbidity in Swedish horses [15], where fetlock lesions were ranked as 1^st^, 11^th^ and 12^th^ most common causes of culling. Lesions in carpal, femoropatellar and tarsocrural joints were also frequent causes of culling. Further, in Hanoverian Warmbloods fetlock, tarsus, back and tendons were the most frequent locations of locomotor apparatus disorders [4]. Present results show that horses with low health scores, previously shown to have lower longevity in a smaller dataset [7], have more clinical findings than other horses already at a young age. The majority of clinical signs seen are joint effusions and lameness. These types of clinical findings are commonly also seen in horses with OC lesions or other developmental disorders. One might suspect that some of these horses coincide. As clinical signs may be due to either local underlying lesions or, on the other hand, to complex compensatory overloading, the need for whole body health examinations is emphasised. The validity of the flexion test is presently debated due to lack of standardisation in amount of force applied and time of provocation [13], and because most clinically sound horses can exhibit minor flexion test reactions depending on intensity of provocation [16]. However, present flexion test results, especially moderate/severe reactions, were highly related to number and severity of PALP findings. Further, it had a large influence on the H2 score, which is related to future longevity [7], suggesting the usefulness of flexion tests.

Previous studies have found a lower risk of early culling, i.e. termination of life, in females compared to males [2; 7]. In present results, females had significantly more clinical findings and lower overall health score, suggesting health status and culling to be two separate traits with a possible difference that mares have an alternative career as broodmares. The results are partly supported by a study of Dutch Warmbloods, where mares had a higher risk of culling from basic dressage. However, no gender differences were found in elite dressage or basic/elite jumping [17]. Similar to the present study, a study of flexion test reactions of 100 clinically sound horses indicated that mares obtained significantly more post flexion test reactions compared to geldings [16]. This may suggest mares to be less resistant to the provocation or more prone to show pain reactions. Indications were found that 5-year-old broodmares had almost twice as high risk of exhibiting sore back muscles/spinous processes compared to 4-year-olds, which was also true if comparing to 4-year-old mares only. This might be caused by the strains of pregnancy, or due to a relatively sudden increase in training after weaning the foal, where training intensity is based more on age than on the actual training level of the horse.

## Conclusion

The health examinations from the RHQT have increased the knowledge of the general health status of young SWB horses, including relevance of separate clinical findings. Obtained results may serve as benchmarking for health status in young riding horse populations, and for assessment of future longevity of horses in connection to insurance and sales examinations, besides as basis for advice on preventive actions to keep horses sound. Further, the data could be used for genetic evaluation of health traits, or as basis for further studies on clinical relevance of separate clinical findings on longevity. However, the significant effect of event emphasises the need for uniform regimes of examination of horses among veterinarians, not only for research purposes but also for the general horse sector as insurances and sales are based on these types of examinations.

## Endnotes

^a^The MathWorks Inc., Natick, Massachusetts, U.S.A.

^b^SAS institute Inc., Cary, NC, U.S.A.

## Abbreviations

H1: Overall score, health examination 1 (medical and hooves); H2: Overall score, health examination 2 (orthopaedic); HOOF: Hoof examination; LOCO: Locomotion examination; MED: Medical examination; PALP: Palpatory orthopaedic examination; RHQT: Riding horse quality test; SWB: Swedish Warmblood studbook

## Competing interests

All authors declare that they have no competing interests.

## Authors’ contributions

All authors contributed to the planning, data evaluation and writing the article. LR and LJ executed the digitalisation of data used in the study. All authors read and approved the final manuscript.

## Supplementary Material

Additional file 1 Percentage of overall scores given for health examination 1 (H1) and 2 (H2), and mean overall score and standard deviation (s.d.) among 8,281 studied horses. Click here for file

Additional file 2 Mean sum of clinical findings, including severity, of medical health (MED), hoof examination (HOOF), palpatory orthopaedic health (PALP), locomotion examination (LOCO) and overall score of health examination 1 (H1) and 2 (H2), during 1983–2005. Click here for file
