# Supplementary figures and images for: Prevalence of clinical findings at examinations of young Swedish warmblood riding horses
Source: Acta Vet Scand. 2013 Apr 18;55(1):34. doi: 10.1186/1751-0147-55-34 (PMC3764978; doi:10.1186/1751-0147-55-34)

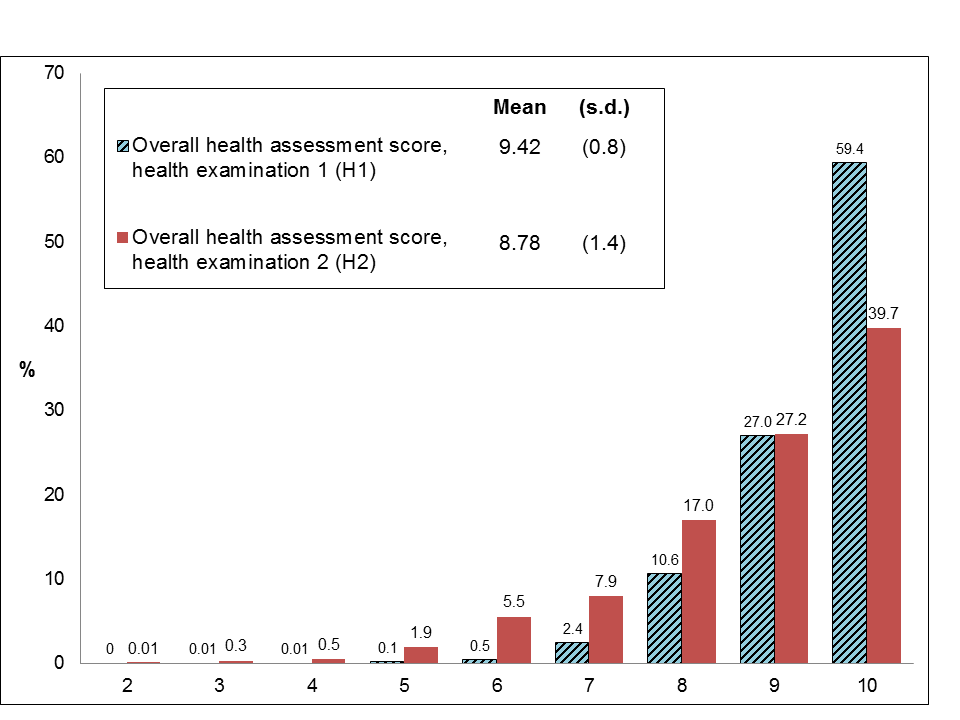

Supplement: Additional file 1 — Percentage of overall scores given for health examination 1 (H1) and 2 (H2), and mean overall score and standard deviation (s.d.) among 8,281 studied horses. [file 1751-0147-55-34-S1.png]

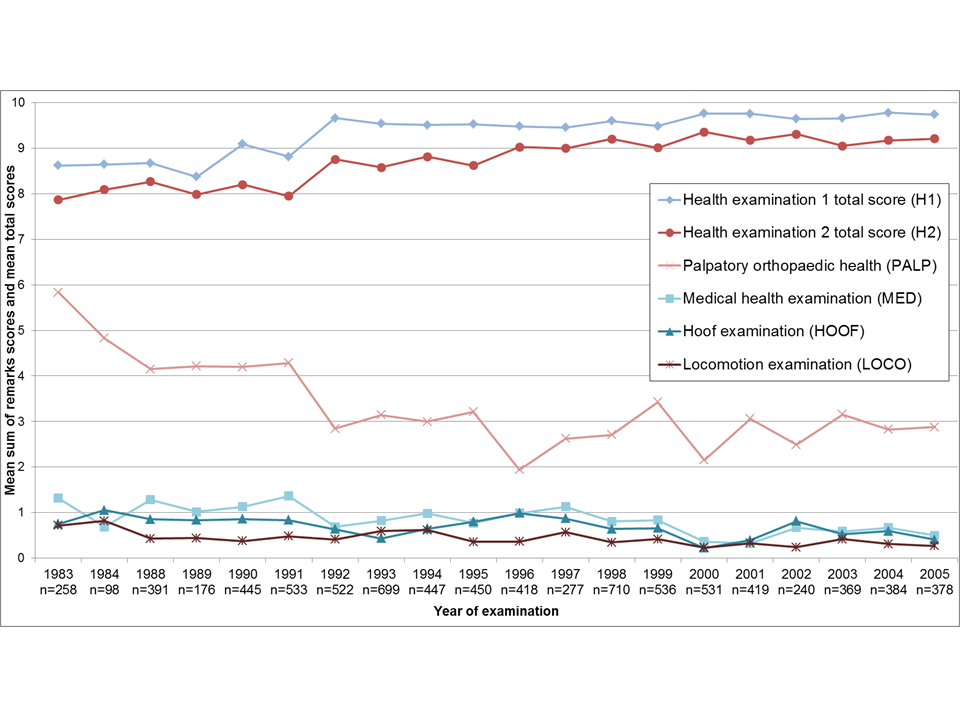

Supplement: Additional file 2 — Mean sum of clinical findings, including severity, of medical health (MED), hoof examination (HOOF), palpatory orthopaedic health (PALP), locomotion examination (LOCO) and overall score of health examination 1 (H1) and 2 (H2), during 1983–2005. [file 1751-0147-55-34-S2.png]
